# The Hull Royal Infirmary

**Published:** 1893-03-04

**Authors:** 


### The Institutional Workshop.

### WITHIN THE HOSPITALS.

### THE HULL ROYAL INFIRMARY.

By Our Special Commissioner.

This institution has existed for more than a century, it having been established in 1782, and then reorganised and extended in 1887. It is interesting to note that, like other provincial institutions, as the town where it is situated has grown and prospered, so the infirmary has grown and prospered too The work was begun in temporary premises on September 26th, 1782, a new infirmary being built and completed two years later at a cost, including the site, of £4,216. In those days people had a knack of building beyond the immediate requirements of the population, and so for nearly thirty years portions of the buildings were not occupied by the patients, or even furnished. As originally constructed the building was planned as a single corridor pavilion, and constituted one straight block, with a corridor at the rear. Mr. George Pycock was the first architect, and his original plan shows a much better appreciation of sanitary and hygienic requirements than those of his successors. In 1839, 1858, and 1863, additions were made to the original buildings, which provided further accommodation, but tended to materially alter for the worse the structural qualities of the buildings, having regard to the uses to which they are put. We have no particulars of the cost of these additions, which must have been considerable, but they certainly did not tend to improve the infirmary. In 1882, at the ninety-ninth annual meeting, it was determined to celebrate the centenary, and with this object it was determined to raise the invested funds by £20,000 and the subscription list by £2,000 a-year. Although this appeal was only partially successful, it was determined to expend £30,000 in increasing the site and erecting thereon new buildings from the plans of Mr. Saxon Snell. The foundation-stone was laid by the Duke and Duchess of Edinburgh on October 1st, 1884. The new wing was opened by the Lord Lieutenant of the East Riding (Lord Herries) at the end of 1885, when more than the £20,000 had been subscribed. It is worthy of notice that the Chairman of the hospital, Mr. Henry Simpson, became interested in the infirmary so long ago as 1840, when he gave as a schoolboy his first donation to the Bazaar Fund in aid of the extensions made in that year. In connection with the Duke of Edinburgh's visit, the Hull General Infirmary became the Hull Royal Infirmary, by which name it is now known and widely appreciated throughout the East Riding. When we visited the infirmary, we were much pleased with the present management, and found that the structural imperfections are largely neutralised by the zeal and energy of the resident staff.

#### Management.

Throughout the whole infirmary there is evidence of the presence of good and intelligent administration. The baths are excellent, but it is to be regretted that zinc slop-sinks are permitted, seeing that they never look cleanly, and that zinc, as a matter of fact, is ill adapted to hospital uses. There can be no question that all slop-sinks should be made of porcelain, and that they should be surrounded by encaustic tiles. The wards are comfortably furnished in regard to chairs, lockers, and bedsteads. The beds, however, leave something to be desired. We were informed that an order had been given to replace those in use by spring mattresses, and to substitute hair for flock in several of the wards. We were surprised to find that flock beds were admitted at all into an institution which has made such sound progress as the Hull Infirmary has done during recent years. There was an abundance of flowers and pictures, which gave the wards an attractive appearance, and the windows at the end of each of the main wards were filled in with some washable material in imitation of stained glass. These windows gave a very handsome appearance to the wards, and if the material wears, as it appears likely to do, it is to be hoped that it will be generally adopted by hospital authorities. Evidence of good management was present throughout the housekeeping and domestic departments of the stores. The kitchen, which is one of the best proportioned, most airy, and well-arranged kitchens we have ever seen, is fitted with J. Salter and Co.'s gas cooking apparatus, than which nothing is better adapted to large establishments. The kitchen, larder, store room, beer cellar, and smaller offices are all situated at the top of the main building, and they exhibit evidence of skilful management and economical arrangement. We were sorry to see that some of the servants, who had otherwise comfortable quarters, had to sleep four in one room, and that the rooms which contained more than one bed, both in this part of the hospital and also in the nurses' home, were not provided with screens. Surely this fact has only to be made known to the ladies of Hull to ensure an immediate supply of the six or a dozen screens that are required to secure the necessary privacy to both nurses and servants.

#### Nurses' Home.

The building now used as a nurses' home was originally the infectious block. It contains a sufficient number of single rooms, each of which is provided with a fireplace, to give a separate room to all but four members of the nursing staff. The dining-room and the nurses' sitting-room are well proportioned and comfortable apartments, but the latter contains no easy chairs. Ordinary bentwood arm-chairs have been supplied in the sitting-room, a class of chair very suitable for a men's smoking-room, but not comfortable enough for nurses to occupy who have been engaged hard at work the whole day in a hospital ward. There is at present no nurses' library, a fact which we commend to the attention of some of the wealthier governors of the Hull Infirmary, in the belief that the fact has only to be stated to secure the necessary supply of books and periodicals. We noticed also that the nurses' beds are not as comfortable as they might be, and we would suggest the substitution of How's spring beds, or some variety of wire wove mattress, throughout the nurses home. The consulting surgeon, Dr. Lunn, justly remarked that the nursing staff of the Hull Infirmary are rightly regarded as typical of the highest type of nurse, and no one can visit the Infirmary without being struck by their pleasant bearing and marked efficiency. We understand that nurses are trained at the Hull Infirmary as Queen's nurses for the Jubilee Institute. The Lady Superintendent, Miss A. L. Cox, whose work is gratefully remembered at the Bristol General Hospital, is to be congratulated upon the high state of excellence to which she has brought her department. There was evidence everywhere of good feeling and friendliness amongst the members of the resident medical staff, and the Committee are to be congratulated upon having secured the services of such excellent representatives of the medical profession for the positions of House and Assistant House Surgeons. It is reported that there are no less than three nurses' homes in Hull, a fact which surely needs the urgent attention of the more intelligent of the inhabitants in order that by combined action they may secure the establishment of one properly kept and efficient institution, from which they will be able to obtain at all times reliable and thoroughly trained nurses for their private houses when they require them.

The Out-patient Department.

This building, which was erected about ten years ago, consists of a main hall and two consulting-rooms. The site placed at the disposal of the architect by the committee is an excellent one, and we regretted to find it had been so badly utilised for the purpose to which it was put. The consulting-rooms are not in communication, and the members of the medical staff have to take their departure when leaving by the main building. Such an arrangement is most undesirable, and should never have been permitted. It seems impossible now to remedy the faults which are so patent in these buildings. We would suggest, however, that some members of the governing body should take an opportunity to visit the new out-patient department at Leeds, and also that at the Great Northern Central Hospital in London, that they may see for themselves how easy it is to facilitate out-patient work by providing buildings upon a plan which readily adapts itself to the efficient and rapid treatment of the patients who resort to the out-patient department of a hospital.

